# Delivering adjuvant and neoadjuvant treatments in the early stages of hepatocellular carcinoma

**DOI:** 10.1080/17474124.2024.2419519

**Published:** 2024-10-22

**Authors:** Bernardo Stefanini, Giulia F. Manfredi, Antonio D’Alessio, Claudia A.M. Fulgenzi, Nichola Awosika, Ciro Celsa, Mario Pirisi, Cristina Rigamonti, Michela Burlone, Federica Vincenzi, Rosalba Minisini, Alessandra Gennari, Vincent Yip, Sarah Slater, Karim El-Shakankery, Ananya Jain, Francesco Tovoli, Fabio Piscaglia, Duncan Spalding, Madhava Pai, David J. Pinato

**Affiliations:** a Department of Surgery & Cancer, Imperial College London, Hammersmith Hospital, London, UK; b Department of Medical and Surgical Sciences, University of Bologna, Bologna, Italy; c Division of Internal Medicine, Department of Translational Medicine, University of Piemonte Orientale, Novara, Italy; d Division of Oncology, Department of Translational Medicine, University of Piemonte Orientale, Novara, Italy; e Section of Gastroenterology and Hepatology, Department of Health Promotion, Mother and Child Care, Internal Medicine and Medical Specialties, PROMISE, University of Palermo, Palermo, Italy; f Barts and the London HPB Centre, Royal London Hospital, Whitechapel, UK; g Edinburgh Cancer Centre, Western General Hospital, NHS Lothian, Edinburgh, UK; h Division of Internal Medicine, Hepatobiliary and Immunoallergic Diseases, IRCCS Azienda Ospedaliero-Universitaria di Bologna, Bologna, Italy; i Hepatobiliary Surgery, Imperial College London and Imperial College NHS Trust, Hammersmith Hospital, London, UK

**Keywords:** Immunotherapy, adjuvant, neoadjuvant, immune checkpoint inhibitors, hepatocellular carcinoma

## Abstract

**Introduction:**

Hepatocellular carcinoma (HCC) presents a formidable challenge in oncology, demanding innovative treatment approaches. Both adjuvant and neoadjuvant therapies, thanks to the introduction of immunotherapy, have emerged as promising strategies in the management of HCC, aiming to reduce the risk of relapse and ultimately to improve survival.

**Areas Covered:**

This review considers current evidence, ongoing clinical trials, and future strategies to elucidate the evolving landscape of neoadjuvant and adjuvant treatments in HCC.

**Expert Opinion:**

Both adjuvant and neoadjuvant regimens, notably those incorporating immune checkpoint inhibitors, demonstrated encouraging safety profiles and efficacy outcomes in HCC.

While significant challenges persist, including optimizing patient selection and endpoint definition, the evolving landscape of neoadjuvant and adjuvant therapy holds promise for maximizing the therapeutic potential of immunotherapy across all stages of HCC. Further insights into tumor biology and host immunity will shape the role of these approaches which are close to becoming reality in clinical practice.

## 1. Introduction

Hepatocellular carcinoma (HCC) is a global health problem affecting nearly one million people per year and is the second most common cause of premature death from cancer, being responsible for more than 800.000 deaths worldwide [1].

Although HCC benefits from the existence of a surveillance screening program [2], only a minority of patients diagnosed with hepatocellular carcinoma can hope for a cure of their disease.

In fact, among the available treatments for HCC, only liver transplantation (LT), liver resection (LR) and ablative techniques like radiofrequency (RFA) or microwave ablation (MWA) are considered curative.

Despite technological advancements and a better patient selection, curative therapies including LR, RFA and MWA achieve 5-year survival rates of 40–70% across different treatment modalities [3].

High recurrence rates, usually within 5 years after the initial treatment, hinder overall survival, with rates of recurrence ranging from 42% to 70% for LR [4,5], 58% to 81% for RFA [6,7] and 65% to 81% for MWA [8,9].

LT outperforms all other treatment options for HCC with a 5-year overall survival rate estimated between 68.1% and 73.2% depending on selection criteria. However, LT is less frequently available for HCC patients. This is due to a variety of factors including staging characteristics at presentation, patient comorbidities and shortage of donors. Even when LT is feasible, relapses can occur in 15–20% of patients within the first five years [9,10].

In order to improve the overall prognosis of HCC patients, two key objectives must be pursued. The first is to maximize the number of patients suitable for and able to undergo curative treatments. The second objective is to potentiate the efficacy of radical therapies for those receiving them, primarily through introduction of novel treatments capable of lowering the risk of relapse and resultant mortality.

Mirroring the observed experience from other solid tumors, a variety of approaches have been developed in recent years including the introduction of adjuvant and neoadjuvant treatments.

Adjuvant treatment is defined as the administration of additional systemic or local therapy (i.e. radiation) in a deferred temporal sequence to the primary anti-cancer therapeutic strategy with the overall intent of reducing the risk of relapse and improving patients’ survival from cancer. The first report of the use of adjuvant therapy dates to 1958 where triethylenethiophosphoramide and nitrogen mustard were tested after gastric cancer resection [11].

Conversely, neoadjuvant treatment is defined as the administration of either systemic or local therapeutic agents prior to definitive curative treatment. There are multiple benefits that can be derived from the use of neoadjuvant therapy including improvement of surgical outcomes by downsizing the disease therefore allowing less demolitive surgery. Another key advantage is to be found in the earlier exposure of a treatment-naïve tumor to cytotoxic, targeted or immune-based therapies. The lower genomic complexity and immune-tolerogenic contexture of the tumor microenvironment that sets apart early-stage cancers from more advanced forms of the disease is postulated to lead to deeper and more durable responses than those observed in more advanced stages. Moreover, neoadjuvant treatment also aims to precociously treat micrometastases, which are thought to play a central role in the development of subsequent disease relapse [12].

Recently, neoadjuvant studies testing novel therapies like immune checkpoint inhibitors (ICI) have provided valuable data on their mechanism of action by examining changes in the tumor microenvironment and peripheral immune responses before and after exposure to ICI [13,14].

Following on from the remarkable advances in the use of immunotherapy in advanced/unresectable HCC, increasing research efforts have been devoted to understanding the role for adjuvant and neoadjuvant therapies in HCC.

**Figure 1.**
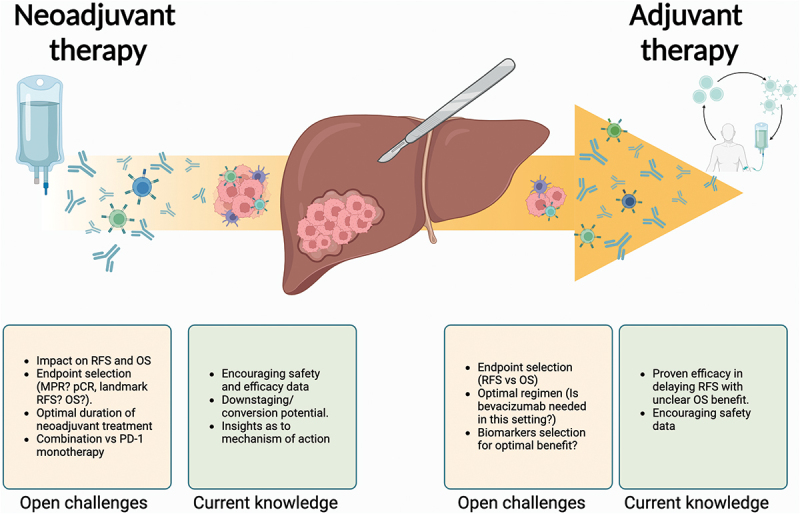
Pending questions and current evidence on the use of neoadjuvant and adjuvant strategies in HCC.

In this review we draw on current available evidence to discuss the rationale for both adjuvant and neoadjuvant therapies in early-stage HCC (Figure 1). Alongside focusing on the indications, benefits and challenges arising from adjuvant and neoadjuvant treatment approaches in HCC, we will specifically highlight the role of ICI in these settings, mindful of the remarkable benefits ICI has provided in unresectable HCC.

## 2. Adjuvant treatment in HCC

The search for effective adjuvant therapies in HCC has been considered an area of significant unmet need for some time. Unlike other tumors, where the development of adjuvant treatments followed evidence of efficacy in advanced/metastatic settings, several clinical trials exploring the utility of adjuvant therapies in HCC were conducted far before sorafenib was identified as an effective systemic anti-cancer treatment (SACT) for advanced disease, in 2007 [15]. Another unique characteristic of HCC, distinct from other solid tumors, is the common interplay between cancer and underlying liver cirrhosis. Cirrhosis poses a dual challenge: it is a competitive risk factor for death and it limits the safe delivery of SACT. This limitation is primarily due to the functional impairment of the liver. This is particularly relevant when considering adjuvant treatments; the coexistence of cirrhosis and cancer may narrow the intended therapeutic index and increase the risk of long-term or life threatening SACT toxicity in potentially curable patients.

Intrinsic resistance to cytotoxic chemotherapy and the lack of highly prevalent therapeutically actionable drivers from a molecular standpoint [16] are recognized challenges to therapeutic drug development in HCC. These aspects are certainly relevant in a metastatic setting, but they are even more important in the adjuvant setting, where the absence of molecular knowledge and the absence of biomarkers able to predict a response severely impair the ability to rationally select agents for adjuvant therapy unlike non-small cell lung cancer [17], melanoma [18] and breast cancer [19].

While the role of SACT in advanced HCC is now established, the development of adjuvant systemic therapy in HCC still presents formidable challenges. Firstly, the lack of consensus and tumor clonality data hampers a biologically informed definition of early versus late recurrence, a definition traditionally based on an arbitrary cutoff at 2 years. The distinction between early recurrence, possibly stemming from clonal dissemination of the original disease, and late recurrence, more likely indicative of de novo tumorigenesis from surrounding field defect, remains elusive, thus leading to controversy in the assessment of effectiveness of adjuvant regimens which are based on recurrence-free survival.

Additionally, predictors of recurrence in HCC are not well-defined, making it challenging to standardize the relative contribution of each factor in prognostic assessment for individual patient. In fact, although the role for tumor size (≥2 cm or <2 cm) and the role for the number of lesions in predicting higher risk of relapse is better defined, even if mainly based on retrospective studies [20,21]; the predictive role of other factors, such as the grade of the differentiation of the tumor or the microvascular invasion (mVI), are less evident due to the heterogeneity and lack of reproducibility of the Edmonson grading system [22,23] and the uncertainty in diagnosing mVI in absence of histological confirmation.

Lastly, meaningful clinical endpoint selection in the adjuvant setting is particularly challenging due to the presence of multiple confounding factors which play a relevant role both for relapse-free survival (RFS) and overall survival (OS).

### 2.1. Systemic anti-cancer treatment

Several adjuvant therapeutic strategies have been tested throughout the years for their clinical value in reducing the risk of recurrence and increasing chances of cure. The choice of agent has been motivated by either direct anti-neoplastic, immune-modulatory and/or anti-viral properties, reflecting evolving knowledge on the molecular pathophysiology of HCC and clinical evidence from trials in advanced disease. Table 1 summarizes the completed clinical studies that investigated the role of adjuvant SACT following either resection or ablative curative procedures in early stage HCC [24–32].

**Table 1.** List of completed studies involving pharmacological treatment in an adjuvant setting in early stage HCC.

Completed adjuvant treatment in early stages HCC							
Trial and author	Drug (dose) vs control	Phase	Treatment arms	Patients	Primary outcome	Follow-up time	Reference
STORM Bruix et al (Lancet Oncol. 2015)	Sorafenib (800 mg/d) vs placebo	III	2	1114	RFS	8.5 (sorafenib) vs 8.4 (placebo)	[24]
NIVOLVE (no full paper available) Kudo et al	Nivolumab (240 mg q2W, doubled dose after cycle 8 q4w)	II	1	55	1-year RFS	NR	[25]
Chen et al (Ann. Surg. 2012)	IFN alpha-2B (5 MU three times a week for 53 weeks) vs observation alone	III	2	268	RFS	63.8 months	[26]
Hasegawa et al (Hepatology 2006)	uracil-tegafur (300 mg/d for 1 year)	III	2	80	RFS	57.6 months	[27]
Mazzaferro et al (Hepatology 2006)	IFN-alpha- 2B (3 MU three times a week for 48 weeks) vs control group	III	2	150	RFS	45 months	[28]
Yoshida et al. (Hepatology 2011)	Vit.K2 (45 mg/d or 90 mg/d) vs placebo	III	3	548	RFS	NR	[29]
Lee et al (Gastroenterology 2015) POSITIVE STUDY	Cytokine-induced killer cells (CIK) 6.4 × 10^9^ autologous CIK cells, 16 times during 60 weeks vs control group	III	2	226	RFS	40.0 months (CIK-treated patients) vs 36.5 months in the control group	[30]
Okita et al. (J. Gastroenterol, 2014)	Peretinoin (600 mg vs 300 mg vs placebo)	III	3	401	RFS	30.0 months	[31]
ImBrave050 Qin et al (Lancet 2023) POSITIVE STUDY	Atezolizumab 1200 mg+Bevacizumab 15 mg/kg, q3w for 1 year vs active surveillance	III	2	668	RFS	17.4 months	[32]
Wang K et al. (Nat Med, 2024) POSITIVE STUDY	Sintilimab (200 mg q3w for 6 months vs surveillance)	II	2	198	RFS	NR	[46]

IFN: Interferon; MU: million Units; NR: Not reported.

#### 2.1.1. Adjuvant TKI therapy

After the positive readouts of the SHARP and Asia-Pacific Trials in years 2007–2008, which demonstrated sorafenib, a multi-targeted Ras/Raf and VEGF inhibitor, as the first systemic anti-cancer therapy known to improve the survival of patients with advanced HCC [15,33], a strong rationale emerged for testing sorafenib as adjuvant therapy post-resection. The multicenter, phase III RCT STORM recruited 1114 patients with complete surgical resection or local ablations who were assigned to either receive placebo (*n* = 558) or Sorafenib (*n* = 556).

No difference was observed between the median RFS estimate of the placebo group (33.7 months) versus 33.3 months achieved in the sorafenib group [24]. To further analyze this complicated topic and to understand whether a better patient selection of the enrolled patients could have influenced the outcome of the study, some of the authors published an interesting study which attempted to characterize putative biomarkers of response to sorafenib therapy. In this study tumor tissue was collected from 188 patients including 83 receiving sorafenib and 105 receiving placebo. All the samples were analyzed using immunohistochemistry, fluorescence in situ hybridization, gene expression profiling and exome sequencing of 19 known genomic drivers of HCC progression. Results did not establish a definitive benefit for adjuvant sorafenib after balancing for molecular tumor characteristics including mutational profiles or transcriptomic signatures previously known to be associated with improved outcome from sorafenib [34].

Another key learning point generated from the STORM trial stems from the challenges of maintaining patients on active treatment post resection. In the sorafenib arm, duration of treatment (12.5 months) and dose intensity (577 mg per day) were significantly shorter than in the placebo arm (22.2 months and 778.0 mg per day) and significantly reduced to the planned time of 48 months.

Interestingly, unlike other cancers such as EGFR mutant [17] or ALK fusion positive non-small cell lung cancer [35], where TKIs are approved as adjuvant treatment, HCC lacks an easily identifiable molecular target able to identify a subgroup of patients more likely to benefit from TKI. This lack of molecular knowledge correlates with the limited success of TKI as adjuvant treatment for HCC.

#### 2.1.2. Adjuvant immune modulating agents

One of the first approaches tested the use of adoptive cytokine therapy with interferons (IFNs), based on their anti-proliferative abilities documented *in vivo* and *in vitro* across various malignancies including HCC [36]. The concurrent anti-viral activity of IFNs, further strengthened the rationale for adjuvant IFN therapy in the pre-direct antiviral agents era, given that hepatotropic viral infection has traditionally represented a leading etiologic factor for HCC.

After initial promising results for IFN-α and IFN-β in the adjuvant setting, generated from small and highly heterogeneous randomized clinical trials (RCT) [37,38], Mazzaferro *et al* conducted an open-label, randomized study on the role of IFN-α in adjuvant HCC. Following LR, 150 hCV-RNA positive patients were randomized to either receive (IFN-α *n* = 76) or active surveillance (*n* = 74). The study failed to meet its primary endpoint of RFS [28]. In a similar fashion, Chen et al. demonstrated that treatment with IFN-α-2B did not influence postoperative recurrence of 268 patients with virally induced HCC following with surgical resection [26].

A number of alternative approaches were tested after failure of adoptive IFN therapy including the use of peretinoin, a synthetic acyclic retinoid able to suppress tumor growth by inducing apoptosis and differentiation of liver cancer cells in preclinical studies [39], or the use of vitamin K2 following pre-clinical evidence of its ability to inhibit growth and invasion of HCC cells through modulation of several transcriptional factors [40]. Neither of them, unfortunately, succeeded in demonstrating a benefit in RFS in phase III trials [29,31].

The first systemically delivered therapy that has met its primary endpoint in adjuvant studies in HCC is adoptive cellular therapy. Encouraged by positive preclinical data [41], Lee et al used cytokine-induced killer cells (CIK) in an adjuvant setting after curative treatments for HCC.

In this seminal RCT, autologous CIK cell-based immunotherapeutic agent (Immuncell-LC), was manufactured by ex vivo culture of patients peripheral blood mononuclear cells stimulated using interleukin 2 and anti-CD3 antibodies [30].

230 patients were recruited from 5 different Korean centers after receiving either RFA, surgical ablation or percutaneous ethanol injection (PEI). They were randomized 1:1 to receive CIK immunotherapeutic agent (*n* = 115) or not (*n* = 115). 4 patients were excluded (1 among immunotherapeutic and 3 in the control group) after the enrollment for violating inclusion or exclusion criteria.

Patients who received adoptive cellular therapy achieved a notable RFS of 44.0 compared to 30.0 months in patients who received placebo, thus becoming the first ever successful adjuvant trial on adjuvant treatment of HCC. However, it should be mentioned that a significant difference in terms of median tumor size (1.8 vs 2.3 cm, *p* = 0.02) favored patients treated with CIK, only partially hampering the results of this trial [30].

This successful approach tested by Lee *et al* highlighted the value of immunomodulation in HCC, promoting an active interest in the use of immunotherapies as adjuvant agents. Subsequently, anti-PD-L1 – anti-VEGF combinations, and anti-PD-L1 and anti-cytotoxic T Lymphocyte-associated protein 4 (CTLA4) doublets, have both demonstrated efficacy in patients with advanced HCC with a rate of disease control ranging from 60 to 75 in phase III trials further reinforcing the support for ICIs in adjuvant setting.

In the single-arm, phase II prospective NIVOLVE trial, 53 patients received one year of adjuvant nivolumab following successful liver resection or RFA. Results regarding safety were reassuring and in keeping nivolumab‘s known toxicity profiles. Encouragingly, the median RFS was not reached, with the 1-year recurrence rate estimated to be 21.4%, although the study was not designed for comparison. Although inter-trial head-to-head comparisons should not be encouraged, it is interesting to note that this rate was approximately half of that reported in the STORM trial (42% 1-year recurrence rate).[25]

In an interesting study published by Li et al. conducted on 517 patients where only 16.8% of them received adjuvant treatment after curative resection, the authors reported a benefit in median RFS among patients who received any kind of adjuvant treatment compared to those who received none (25.2 months vs 16.1 months). Although intriguing, the results of this trial are hard to generalize mainly due to the imbalance between treated and untreated population, the multiple regimens used and the use of a propensity matching strategy to achieve these results [42].

Following the unprecedented survival benefit demonstrated in metastatic setting (the ImBrave150 trial), a lot of attention has also been directed toward the combination of atezolizumab plus bevacizumab (A+B) in the adjuvant setting. Indeed, the strong rationale behind this combination has been based on the synergistic effects of anti-VEGF and anti-PD-L1 combinations. Bevacizumab, in fact, not only influences tumor angiogenesis, but also enhances anti-cancer immunity by altering the tumor microenvironment [43].

Qin et al published the results of IMbrave 050 trial, a phase III study which involved 668 patients with high-risk for recurrence HCC who underwent either curative resection (*n* = 585) or ablation (*n* = 83). Patients were randomized to either receive the combination of Atezolizumab (1200 mg iv, q3w) plus Bevacizumab (15 mg/kg iv, q3w) for 12 months or active surveillance after the curative treatment [32].

High risk criteria were selected according to the treatment received, for LR high-risk criteria were considered as the presence of 4 or more nodules, up to three nodules but either with the largest one ≥5 cm or with portal vein invasion or poor tumor differentiation, while features for high risk criteria for patients who underwent ablation were either the presence of multiple tumors or a single tumor between 2 and 5 cm. Notably, despite being plausible risk factors for HCC recurrence, these criteria were never properly validated in this setting.

Conversely, patients with metastasis or large vascular invasion involving main portal vein were excluded The primary endpoint of the study was met, with median RFS not being reached in both groups. Patients treated with A+B had a 13% reduction risk of developing recurrence at 1 year and a 28% cumulative risk reduction with an acceptable safety profile.

Notably the benefit in terms of RFS favored the A+B combination in the subgroup of patients who underwent ablation rather than the resection group (HRs for RFS 0.60, 0.75, respectively).

Despite positive data for RFS, no improvement in terms of OS was observed in the 17.4 months of median follow-up time of the study, both the highly immature survival data and the design of the trial which allowed cross-over surely impacted the lack of OS benefit.

Although the RCT is the first to provide evidence of efficacy of adjuvant treatment some uncertainties are partially hindering the clinical impact of the study.

Firstly, we note the high proportion of patients with an HBV-related HCC who are significantly less likely to have underlying liver cirrhosis. Notably the authors did not report the proportion of cirrhotic patients in the cohorts. Secondly, patients with viral related HCC are also considered to be slightly more responsive to the combination of A+B than patients with non-viral etiology as suggested in some subgroup analysis of the IMbrave 150 [44].

It is also important to highlight that 10% of the patients across both groups received TACE as an adjuvant treatment with potential implications related to increase in ICI responsiveness after TACE, in particular considering that such a treatment is not commonly performed in every country as a viable adjuvant treatment.

In addition to this, both the progressive convergence of the two curves of RFS which are overlapping after 18 months of follow up and the absence of a proper validation of the high-risk criteria used to stratify the population also represent relevant concerns.

Furthermore, the inclusion of a considerable amount of patients who had portal vein invasion could significantly impair these findings, in fact the benefit in RFS could be seen as a result of an initial under staging of the tumor rather than an effective adjuvant regimen.

It is important to highlight the author acknowledgment of some of these above concerns, with resultant pre-specified subgroup analysis. These analyses were reassuring, in particular the benefit in median RFS was similar between HBV and other etiologies and it was consistent between patients who received TACE and those who did not, actually favoring the latter.

Despite these concerns, the IMbrave 050 certainly represents the cornerstone of a new paradigm in the treatment of HCC being the first successful phase III study in an adjuvant setting.

To further complicate the interpretation and the clinical applicability of the results of this pivotal phase 3 trial, the most recent update after a prolonged follow-up (35.1 months) demonstrated the lack of a benefit in RFS with the treated cohort who had an HR of 0.90 (95% CI: 0.72, 1.12) while data about OS were still immature to be analyzed but failed at the moment to show any benefit for the treated population [45].

Additional support to the use of adjuvant immunotherapy, a phase II study conducted at 6 Chinese hospitals investigated the use of the anti-PD-2 agent, sintilimab. In this trial, 198 patients who underwent liver resection for hepatocellular carcinoma with mVI were randomized 1:1 to receive 6 months adjuvant sintilimab (*n* = 99) or surveillance (*n* = 99). Patients who received sintilimab had a 46.6% lower risk of relapse or death compared to surveillance. Some imbalances in prevalence of cirrhosis and risk of high-grade mVI were present in the baseline characteristics of the two groups, but the RFS benefit was maintained after adjusting for these factors. However, similarly to ImBrave150, no benefit in OS was observed, even though the trend appeared more favorable (HR 0.51, 95%CI 0.25–1.01) [46].

Key differences among these two trials includes a different patient selection with the latter including only resected patient with mVI, treated for a shorter period of time (6 months versus 12 months) using only a single agent rather than a combination.

In addition, the exclusive Chinese enrollment in the sintilimab study also influences the extent to which these results can be generalized, given the high percentage of non-cirrhotic HBV patients enrolled in this trial. Nevertheless, such differences raise interesting questions around optimal treatment durations, appropriate patient selection, and whether the presence of bevacizumab is really beneficial in this setting.

Several new studies regarding the use of adjuvant ICI are currently being conducted, including Checkmate 9DX (testing nivolumab, NCT03383458), EMERALD-2 (testing durvalumab + bevacizumab, NCT03847428) and KEYNOTE-937 (Pembrolizumab, NCT03867084). Data emerging from these studies will help to clarify the role of ICI in the adjuvant setting. A summary of all relevant ongoing studies is displayed in Table 2 [47–61].

**Table 2.** List of ongoing clinical trials involving adjuvant treatment using immune checkpoint inhibitors.

Ongoing clinical trials on adjuvant treatment with ICI in early stages HCC*						
NCT Number/trial name	Drug(s)	Endpoint	Phase	Arms	End of trial	Ref
NCT05240404	Toripalimanb	DFS (primary)	II	2	July 2024	[47]
NCT03859128 JUPITER 04	Toripalimanb	RFS	II–III	3	August 2023	[48]
NCT04639180	Camrelizumab + Apatinib	RFS	III	2	Jul 2024	[49]
ChiCTR2200063003 TIDE	Tislelizumab+donafenib+TACE	RFS	III	1	December 2024	[50]
NCT 03847428 EMERALD-2	Durvalumab ± Bevacizumab	RFS	III	3	August 2025	[51]
NCT03867084 KEYNOTE-937	Pembrolizumab	RFS	III	2	August 2029	[52]
NCT 03383458 Checkmate9DX	Nivolumab	RFS	III	2	December 2025	[53]
NCT03630640	Nivolumab	1-year local recurrence rate	II	1	Dec 2023	[54]
NCT05111366 ALTER-H006	TQB2450 (Anti-PD-L1)+ Anlotinib	1-year RFS	II	1	May 2024	[55]
NCT05407519	Tislelizumab + Sitravatinib	2-year RFS	II	1	June 2026	[56]
NCT05367687	Camrelizumab±Apatinib	RFS	II	2	April 2026	[57]
NCT06059885	Tislelizumab + TKI	Tumor recurrence rate	II	2	December 2025	[58]
NCT05564338	Sitravinib + Tislelizumab	RFS	III	4	April 2028	[59]
NCT05489289	AK104 (Anti PD-1)	RFS	III	2	November 2026	[60]
NCT05545124	Donafenib ± Tislelizumab	1-year RFS	II	1	November 2024	[61]

* This table included only active trials designed to assign adjuvant treatment involving at least a single ICI combined or not with TKI. Trials investigating multiple cancers types were excluded, trials involving advanced stage were excluded. Trials involving combination of loco-regional treatment (HAIC, TACE, etc.) and ICI were also excluded. Some of the dates are antecedent to the date of publication, in these case every trial was manually checked and results were not reported at the moment of submission.

RFS: Relapse-free survival; DFS: Disease-free survival.

### 2.2. Local treatments

While the focus of adjuvant therapy has been mainly centered around use of SACT after primary curative therapy, it should be acknowledged that several studies evaluated a wide array of non-systemic adjuvant treatment options, including TACE [62,63], Hepatic Artery Infusion Chemotherapy (HAIC) [64,65], Internal Radiation Therapy (IRT) [66], and stereotactic body radiation therapy (SRT) [67]. Several studies aimed to establish the impact of loco-regional techniques as adjuvant treatments, with interesting results from the RAISE trial recently presented at ASCO-GI 2024. In this phase II, multicentre RCT conducted in 6 Chinese sites, 148 hCC patients were randomized to receive either intensity modulated radiation (*n* = 74) therapy or active surveillance (*n* = 74), following hepatectomy and narrow margin of resection (≤1 cm). Results were stratified for tumor size (≤5 vs. >5 cm) and presence of mVI. The primary endpoint of RFS was met, demonstrating that 78.4% of the patients treated with radiotherapy achieved RFS at 2 years, compared to 57.4% in the surveillance arm [68].

Li et al demonstrated in a RCT that adjuvant HAIC was able to improve DFS (20.3 months vs 10.0 months) in a cohort of 315 patients with mVI. Significantly, the large majority of patients were HBV positive (around 87% for both arms) and both AFP and des-gamma-carboxy prothrombin were unfavorable for the control arm [69].

The use of adjuvant TACE and IRT has also been studied, including a large multicenter, retrospective study with matched controls [70], and a comprehensive network metanalysis by Ye *et al* (including 5723 patients from 32 RCTs) [71]. Both studies concluded that among all patients who received adjuvant local treatments post-resection, only those who subsequently underwent TACE showed an improved 1-year OS; in contrast, only IRT improved 3-year OS. In contrast to this, a similar study including 1927 patients receiving adjuvant TACE, reached different conclusions, despite considering similar patients in terms of geographical origin and stage [72].

It should be considered that these findings were achieved in the context of extremely heterogeneous populations within the meta-analyses. Many studies included were retrospective in nature, and the few included prospective studies rarely stratified according to known risk factors for recurrences, such as tumor size, presence of mVI, or underlying cirrhosis. The high proportion of non-cirrhotic patients recruited to some of these studies (as high as 86%) [73], alongside a higher propensity to perform liver resection even in the presence of MVI may severely influence meta-analyses findings. Furthermore, as the large majority of the included studies were entirely conducted in China and Japan, the overall generalizability of the results also represent a concern.

In conclusion despite vast amounts of data, predominantly coming from retrospective studies, heterogeneity has significantly impacted the generation of universal consensus over the use of these therapies, with Chinese guidelines [74] conflicting over Western International societies (AASLD, EASL, ESMO) [75–77] in recommending adjuvant TACE after liver resection, in patients with high risk of recurrence.

## 3. Neoadjuvant treatment in HCC

Despite concerns regarding the delay or missing of the primary treatment due to severe adverse events, neoadjuvant regimens have established themselves as the standard of care treatment in patients with lung cancer and melanoma after demonstrating a benefit compared to placebo or adjuvant therapy alone.

The use of ICI in earlier stages of HCC is certainly supported by significant evidence of its efficacy in other cancer entities. In addition to this, the rationale behind neoadjuvant strategy with ICI is even more sound, in fact due to the mechanism of action of ICI, the neoadjuvant regimen benefits from the presence of the tumor antigen at the time of the ICI administration, conversely to the adjuvant strategy where the tumor is supposed to be fully removed at the time of the drug administration.

Further support for neoadjuvant treatments comes from studies that demonstrated how ICI efficacy may be higher in patients with a lower tumor burden [78,79] than in patients at a more advanced stage of the disease.

Notably, patients with HCC eligible to receive neoadjuvant treatment, therefore amenable to surgery, usually have an excellent liver function and a good performance status which makes them optimal candidate to receive additional treatment and unlikely to develop treatment-related toxicities that might prevent them to undergo radical treatments or any severe consequence from treatment related side effects.

### 3.1. Safety and efficacy of neoadjuvant treatment with ICI

Evidence regarding safety and the efficacy of neoadjuvant immunotherapy in patients with early-stages of HCC is starting to accumulate and it is summarized in Table 3 [80–82].

**Table 3.** List of completed studies on neoadjuvant treatment with ICI in patients with HCC.

Completed studies on neoadjuvant treatment for HCC							
Author	Phase	Drugs	Enrolled patients	Objective response rate %	Major Pathological response	Median RFS (months)	Ref
Marron et al.	II	Cepilimimab	21	15%	20%	NR	[80]
Ho et al.	II	Nivolumab + Cabozantinib	15	7%	42%	NR*	[81]
Kaseb et al.	II	Nivolumab ± Ipilimumab	27	23% (Nivolumab) 0% (Nivolumab+Ipilimumab)	33% (Nivolumab) 27% (Nivolumab +Ipilimumab)	9.4 (Nivolumab) 19.5 (Nivo+Ipilimumab)	[82]

*Authors reported that 5/12 patients treated relapsed in around 1 year of follow-up.

NR: Not reported.

A limited number of early-phase trials have investigated use of neoadjuvant ICI therapy in resectable HCC. Choice of agents include PD-1 monotherapy, ICI doublets and PD-1 plus TKIs (Table 3). Neoadjuvant treatment durations are typically shorter (6–8 weeks) than adjuvant studies (1 year), and in some cases, the neoadjuvant phase is later followed by an adjuvant course of ICI to further prevent disease relapse.

In an open-label, phase Ib trial by Ho and collaborators, neoadjuvant nivolumab (4 cycles of nivolumab, given every 2 weeks) and cabozantinib (40 mg once daily, for 8 weeks) proved to be effective in 15 patients with locally advanced HCC. None of the treatment-related adverse events (TrAE) observed precluded surgery, hence the study reached its primary outcome of feasibility. Out of 15 enrolled patients with locally advanced HCC, 12 underwent surgical resection (1 died from infection, 1 refused surgery and 1 had disease progression); 42% achieved a major pathological response (MRP; defined as ≥ 90% tumor necrosis), with one patient achieving complete pathological response [81]. Notably, 42% of the patients enrolled in the trial developed a relapse after a median follow-up of approximately 1 year. Such an elevated percentage is almost certainly related to the inclusion of patients who were not originally amenable to surgical resection.

In a phase II, single-arm trial published by Marron *et al*, 21 patients with resectable HCC received two doses of cemiplimab, followed by curative surgical resection. Patients subsequently receive eight cycles of additional, adjuvant cemiplimab 3-weekly. The study’s primary outcome was the achievement of a significant tumor pathological response, defined as > 70% necrosis of the resected tumor. MPR was seen in 20% of the analyzed population, paired with a 15% objective radiological response rate according, as per RECIST 1.1 criteria, at the pre-surgical assessment. Treatment was well tolerated, with just 2 patients experiencing a grade 3 TrAE and only one surgical delay of 2 weeks. Interestingly, the authors also reported that among the 7 patients who achieved at least a 50% tumor necrosis at the time of surgery, histological analysis identified a higher density of tumor-infiltrating lymphocytes and an increased density of CD8 T cells within the tumor lesion, but not in the adjacent areas. Remarkably in ≥ 50% necrosis achievers, the immune infiltrate significantly increased between the baseline and post-resection analysis, whereas this was not the case in patients who did not achieve significant necrosis [80].

Kaseb and colleagues published the result of a similar study, where 27 enrolled patients received 240 mg of nivolumab 2-weekly ±1 mg/kg of ipilimumab neoadjuvantly. This was followed by surgical resection and an adjuvant phase including either just nivolumab (480 mg nivolumab, 4 weekly for 2 years) or nivolumab +1 mg/kg of ipilimumab (6 weekly for up to four cycles) [82]. Results observed were similar to previous, with a MPR of 33% and 27% in the nivolumab and nivolumab + ipilimumab groups, respectively. Additionally, there was a relative increase in the percentage of ≥Grade 3 TrAE in the group of patients receiving doublet ICI (43%), which is not uncommon following administration of dual checkpoint inhibitor blockade. Twenty-six percent of patients did not receive surgery but despite the above, no surgical cancellations were considered to be secondary to TrAEs; 4 out of 27 patients developed disease progression, therefore confirming interval disease progression as a significant risk associated with delivery of neoadjuvant treatments.

Similarly to the study by Marron et al, the authors observed a higher immune cell infiltration in patients achieving a major pathological response, in both single and doublet ICI cohorts. Interestingly, however, they noticed how the increase in immune cell infiltration after resection was present only in the cohort of patients achieving pathological response and treated with doublet ICI, and not in patients who received nivolumab alone, irrespective of the patient’s pathological response. Such an observation may indeed provide further rationale supporting the use of combined treatment.

Encouragingly, further evidence supporting PD-1/CTLA-4 blockade has been highlighted by D’Alessio et al., who reported the preliminary results of PRIME-HCC [83], a phase Ib study of neoadjuvant ipilimumab (1 mg/kg, day one only) and nivolumab (3 mg/kg, day 1 and day 22) [84].

Of note, different from the previous studies, patients enrolled in PRIME-HCC received only neoadjuvant treatment and no peri- or post-surgical treatment. The trial was also designed to involve only patients amenable to upfront surgery. Among the 17 patients enrolled at the time of the publication of the abstract only 1 of them (6%) had a surgical delay (unrelated to ICI toxicity) thus demonstrating an acceptable safety profile. Notably the authors reported a pathological complete response (pCR) rate of 22% at the surgical evaluation, and although not designed to evaluate RFS, only one patient relapsed after ICI treatment, going on to achieve partial response to first-line treatment with A+B in the advanced setting. The full publication of this study will undoubtedly be very informative, being the only study involving strictly neoadjuvant treatment in early-stage HCC patients operable from diagnosis.

Another intriguing study was recently published by Li et al. who conducted the NOTABLE-HCC, a Phase 1b trial on the use of neoadjuvant tislelizumab plus SRT in early stage of HCC followed by an adjuvant treatment with tislelizumab. The result of this study confirmed the optimal safety of the neoadjuvant approach with no surgery delay reported among the 20 patients enrolled in the trial. Notably, the authors also provided translational analysis demonstrating the increase in both adaptive and innate immune cells in patients treated with neoadjuvant regimen [85].

Han and colleagues examined the data from the 4 studies cited in a metanalysis including 88 patients who showed a pooled MPR and complete pathological response of 0.23 (95% C.I. 0.14-0.-36) and 0.19 (95% C.I. 0.10–0.30), respectively [86]. Zhao et al. included in the analysis also patients with higher stages of HCC pooling data from 193 patients reaching similar conclusion both in terms of MPR 0.27. (95%CI, 0.15–0.39.) and pCR 0.13 (95%CI, 0.7–0.19) [87].

### 3.2. Systemic therapy as a downstaging or conversion therapy

Besides its intriguing role in the management of early stages HCC prior to receiving surgical or loco-regional treatment, immunotherapy is also the focus of research efforts as a possible downstaging option, given the significant proportion of patients who are diagnosed at an advanced stage. However well-designed clinical trials are lacking, therefore current evidence is mainly dependent upon observational studies.

In this setting, patients are treated with ICI with the aim to induce significant tumor downstaging to later allow curative treatment options which were previously unfeasible, differently from the neoadjuvant approach where treatment is given only to patient who are resectable upfront.

For HCC patients the aim of downstaging is usually to allow liver transplantation [88]; however, the use of ICI in transplant candidates patients is still heavily debated due to initial reports showing high risk of organ rejection in patients treated with ICI [89,90].

Thanks to a more judicious management of ICI in this setting including careful titration of immune suppression and adoption of more stringent follow-up post ICI dosing [91,92], updated data from observational studies were reassuring, although no solid evidence in favur of ICI-LT integration is currently available [93].

In addition, one must consider that besides the wash-out period from ICI, several factors are probably connected to the development of graft rejection, including factors related to the immunomodulatory agent (i.e. dosage, mechanism of action, number of administrations) and factors related to the patient (i.e. etiology of underlying liver cirrhosis, tumor microenvironment, etc.).

In 2023, the European Society of Organ Transplantation (ESOT) published a consensus report on HCC downstaging, where they encouraged consideration of downstaging treatments for all patients diagnosed with HCC without extra-hepatic disease or macrovascular invasion, including those beyond transplant criteria [94]. However, within the same paper, the authors were unable to provide any conclusive recommendations or conclusions on the safety of ICI prior to liver transplant, due to insufficient published evidence.

Several trials are currently recruiting with highly anticipated results regarding this topic, as summarized in Table 4 [84,95–105]. It is expected that data emerging from these studies will help clarify the potential benefit of ICI in the early-stage therapeutic algorithm.

**Table 4.** List of ongoing trial on the use of ICI as neoadjuvant treatment in patients with HCC.

Ongoing clinical trials on neo-adjuvant*							
NCT Number/Trial Name	Drugs	Arms	Stage	Primary Endpoint(s)	Phase	End of trial	Ref
NCT03682276 PRIME-HCC	Nivolumab+Ipilimumab	1	Resectable HCC	Delay to surgery, Safety	I-II	Dec 2023	[84]
NCT04721132	Atezolizumab + Bevacizumab	1	Resectable HCC	Safety, pathological complete response	II	Dec 2027	[95]
NCT04850040	Camrelizumab+Apatinib+Oxaliplatin	1	Potentially resectable HCC	MPR (≤10% residual viable tumor)	II	Dec 2024	[96]
NCT03510871	Nivolumab+Ipilimumab	1	Potentially eligible for curative surgery	ORR (10% tumor shrinkage)	II	Dec 2026	[97]
NCT05471674	Anti-PD-1	1	Borderline resectable	% of patients achieving ≥ 30% tumor necrosis	II	Dec 2022	[98]
NCT05908786 MORPHEUS-NEO HCC	Atezolizumab+Bevacizumab+ Tiragolumab+Tobemstomig	4	Resectable	MPR (≤10% residual viable tumor)	I-II	Dec 2026	[99]
NCT05440864 NEOTOMA	Durvalumab+Tremelimumab	1	resectable	Safety	II	Nov 2026	[100]
NCT03867370	Toripalimab ± Lenvatinib	4	Resectable	MPR and pCR	Ib-II	Oct 2022	[101]
NT05389527 NeoLeap HCC	Pembrolizumab ± Lenvatinib	1	Resectable	MPR (defined as ≥ 50% necrosis)	II	July 2025	[102]
NCT05807776	Tislelizumab ± Lenvatinib	2	Resectable	MPR rate (tumor necrosis ≥ 70%)	II	Dec 2025	[103]
NCT04888546	TQB2450 (anti-PD-L1)+ Anlotinib	1	Resectable	pCR	I-II	July 2024	[104]
NCT04727307	Atezolizumab+ Bevacizumab	2	Resectable (prior to RFA)	RFS	II	July 2027	[105]

* This table included only active trials designed to assign neoadjuvant treatment involving at least a single ICI combined or not with TKI. Trials investigating multiple cancers types were excluded, trials involving advanced stage were excluded. Trials involving combination of loco-regional treatment (HAIC, TACE, etc.) were also excluded.

Some of the dates are antecedent to the date of publication, in these case every trial was manually checked and results were not reported at the moment of submission.

MPR: Major pathological response; ORR: Objective response ratio; pCR: Complete Pathological Response; RFS: Relapse-free survival.

## 4. Expert opinion

Two separate RCTs demonstrated a benefit in RFS after curative treatments for HCC; however, neither of them has shown an improvement in OS up to now.

Although the evidence provided by these trials is certainly solid, some aspects hinder their applicability in clinical practice, in particular for patients with cirrhosis. Aside from the specific issues related to the design of both trials, the pivotal question to be answered is whether RFS represents a suitable endpoint to be used in the adjuvant setting to instigate a change in clinical practice.

In order to be clinically significant, the benefit in RFS must be sustained, a characteristic that was not met by adjuvant therapy with A+B in IMbrave050 trial [32]. In fact, if we imagine OS as a sum of RFS and post-relapse survival (PRS), we can understand that a time limited benefit in RFS will hardly represent an effective surrogate for OS on these patients who are likely to have a considerably long PRS.

Little data is available regarding PRS, however in the sintilimab trial, around 80% of patients received either TACE or curative treatments for HCC relapses, both of which are expected to provide long PRS, while in only 20% of cases, patients received systemic therapy at relapse.

Additionally, if we look at the reconstructed pooled curves of recurrence-free survival of control arms from STORM and IMbrave050 recently published by Cabibbo *et al* [106], it can be seen that the ‘second wave’ of relapse in untreated patients usually happens around two years from the primary treatment, underlying the need of longer follow-up for adjuvant therapy in HCC.

Given these considerations, if OS cannot be directly considered among the suitable endpoint of adjuvant treatment due to its limitation, including the competitive risk of death from cirrhosis and the impact of subsequent treatments on OS, we must consider possible alternatives. The first is a sustained difference in RFS, which should be large and prolonged enough to eventually impact OS. Another possible outcome could be tumor-related overall survival, which could help to eliminate the bias in longer follow-up caused by death from different causes, therefore limiting the risk of undertreating patients.

More data is required to fully position adjuvant treatment strategies – particularly regarding insight into the required length of RFS benefit necessary to guarantee an impact on OS. We should also aim to understand the best treatment strategy (ICI single-agent vs doublet) and to find a more accurate patient selection, possibly driven by stratifying biomarkers, to be able to better assess the probability of response to treatment and identify patients at higher risk of relapse.

The greatest competitor to adjuvant approaches comes from the use of neoadjuvant therapies. However, compared to adjuvant therapy, evidence supporting neoadjuvant or perioperative ICI is based on small feasibility studies, without solid linkage to key efficacy outcomes of interest. While the ultimate goal in HCC care is to improve OS, alternative endpoints are necessary to facilitate effective development of HCC neoadjuvant therapies, due to the prolonged OS that is typical of patients with HCC, often as a result of effective post-relapse therapy. While a sustained benefit in RFS is a possible approach to identify success from neoadjuvant therapy, the evaluation of pathological responses might identify those patients who will experience a clear benefit from addition of immunotherapy to surgical care. A crucial point that is likely to shape future research in this setting is to understand to what extent pathological regression can be considered as a valid surrogate biomarker, and potentially a primary outcome measure, for neoadjuvant studies.

Lastly, one of the most appealing characteristics of neoadjuvant studies is to provide the opportunity to test cancer biology and its responsiveness to ICI. The amount of data which can be collected from resection specimen is invaluable, and it will probably be essential in the quest for biomarkers predictive of response, helping us to maximize the benefit of ICIs in patients with HCC.

When we consider all data on the use of immunotherapy in early-stage HCC, it seems probable that the best option will involve perioperative treatment with systemic therapy before and after surgery, rather than limiting to a strictly neoadjuvant or adjuvant regimen.

The pairing of neoadjuvant and adjuvant treatments recently demonstrated a significant benefit in melanoma and lung cancer, proving that the addition of pembrolizumab in neoadjuvant setting was able to improve event-free survival in two large phase III trials, where patients were then resected and later treated with adjuvant treatment [107,108].

Interestingly, the majority of the phase II studies published on HCC have already partially adopted this strategy, achieving positive results. Given the small amount of available data, it is not yet possible to identify the most effective regimen or make reasonable assumptions as to whether a perioperative treatment schedule will be more beneficial than neoadjuvant or adjuvant regimens alone. It is however plausible to think that, thanks to the increasing understanding of ICI mechanism of action and interaction with the tumor microenvironment, in the future the majority of patients with HCC both at an advanced stage aiming to downstage, or at an early stage, will be exposed to ICI either in the form of adjuvant treatment, neoadjuvant or both.

Key to success in the delivery of immunotherapy in early-stage disease is a better understanding of the complex interplay between tumor biology and host immunity, a goal that is necessary to achieve to maximize the beneficial effect of an increasing amount of systemic therapeutic options across the various stages of HCC.

## 5. Conclusion

The role of ICI is rapidly expanding in HCC, due to the presence of effective surveillance programs and high rates of recurrence, HCC represents an ideal candidate for neoadjuvant and adjuvant treatment. To date, the efficacy of ICI makes them the most logical drug of choice.

Following positive results from two adjuvant trials, post-operative ICI is closer to become reality. However, at present the role and use of immunotherapy in early stages seems far from established. Without definite OS benefit from adjuvant therapies, better predictive biomarkers, and knowledge around optimal patient selection, adjuvant treatment is still a work in progress.

In addition, several neoadjuvant trials with solid biological rationale are being conducted, with potentially practice-changing implications. We are just at the beginning of understanding the impact neoadjuvant therapies can have in HCC, in terms of efficacy and safety. Data from ongoing studies is eagerly awaited to further clarify the potential impact on patients affected by HCC.

## Article highlights

- Hepatocellular carcinoma (HCC) is burdened by high recurrence rates after curative treatments which significantly influence the survival probability of affected patients.
- Adjuvant treatments following liver resection for HCC are currently under investigation, both systemic anti-cancer therapies and non-systemic treatments are showing interesting results, in particular immune checkpoint inhibitors (ICI) have finally demonstrated efficacy in the adjuvant setting in a phase 3 trial, however the clinical applicability of this result is still debatable.
- Neoadjuvant immunotherapy with ICI is also emerging as a promising tool for early-stage HCC. Trials exploring ICI have demonstrated safety and intriguing efficacy. This approach leverage the immune system’s ability to respond to the tumor’s antigens while they are still present, potentially offering advantages over post-surgical adjuvant therapies.
- Both adjuvant and neoadjuvant strategies are in the pipeline; however, they still have something to prove before entering clinical practice. Further research is needed to establish their impact on long-term outcomes, identify relevant biomarkers, and refine patient selection to optimize treatment responses.

## Abbreviations

CIK: Cytokine-induced Killer
CTLA4: Cytotoxic T Lymphocyte-Associated Antigen 4
EGFR: Epidermal Growth Factor Receptor
HCC: Hepatocellular Carcinoma
HBV: Hepatitis B Virus
ICI: Immune Checkpoint Inhibitors
IFN: Interferon
IRT: Internal Radiation Therapy
LR: Liver Resection
LT: Liver Transplantation
mVI: Microvascular Invasion
MWA: Microwave Ablation
MPR: Major Pathological Response
OS: Overall Survival
pCR: Pathological Complete Response
PD-1: Programmed Cell Death Protein 1
PEI: Percutaneous Ethanol Injection
RECIST: Response Evaluation Criteria in Solid Tumors
RFA: Radiofrequency Ablation
RCT: Randomized Controlled Trial
RFS: Recurrence-Free Survival
SACT: Systemic Anti-Cancer Treatment
TACE: Transarterial Chemoembolization
TKI: Tyrosine Kinase Inhibitors
TrAE: Treatment-related Adverse Events
VEGF: Vascular Endothelial Growth Factor
